# The microbiota–gut–brain axis and neurodevelopmental disorders

**DOI:** 10.1093/procel/pwad026

**Published:** 2023-05-11

**Authors:** Qinwen Wang, Qianyue Yang, Xingyin Liu

**Affiliations:** State Key Laboratory of Reproductive Medicine and offspring Health, School of Public Health, Nanjing Medical University, Nanjing 211166, China; Department of Pathogen Biology-Microbiology Division, Key Laboratory of Pathogen of Jiangsu Province Key Laboratory of Human Functional Genomics of Jiangsu Province, Nanjing Medical University, Nanjing 211166, China; State Key Laboratory of Reproductive Medicine and offspring Health, School of Public Health, Nanjing Medical University, Nanjing 211166, China; Department of Pathogen Biology-Microbiology Division, Key Laboratory of Pathogen of Jiangsu Province Key Laboratory of Human Functional Genomics of Jiangsu Province, Nanjing Medical University, Nanjing 211166, China; State Key Laboratory of Reproductive Medicine and offspring Health, School of Public Health, Nanjing Medical University, Nanjing 211166, China; Department of Pathogen Biology-Microbiology Division, Key Laboratory of Pathogen of Jiangsu Province Key Laboratory of Human Functional Genomics of Jiangsu Province, Nanjing Medical University, Nanjing 211166, China; The Affiliated Suzhou Hospital of Nanjing Medical University, Suzhou Municipal Hospital, Gusu School, Nanjing Medical University, Nanjing 211166, China; Department of Microbiota Medicine, The Second Affiliated Hospital of Nanjing Medical University, Nanjing 211166, China

**Keywords:** neurodevelopmental disorders, gut microbiome, microbiota, gut, brain axis

## Abstract

The gut microbiota has been found to interact with the brain through the microbiota–gut–brain axis, regulating various physiological processes. In recent years, the impacts of the gut microbiota on neurodevelopment through this axis have been increasingly appreciated. The gut microbiota is commonly considered to regulate neurodevelopment through three pathways, the immune pathway, the neuronal pathway, and the endocrine/systemic pathway, with overlaps and crosstalks in between. Accumulating studies have identified the role of the microbiota–gut–brain axis in neurodevelopmental disorders including autism spectrum disorder, attention deficit hyperactivity disorder, and Rett Syndrome. Numerous researchers have examined the physiological and pathophysiological mechanisms influenced by the gut microbiota in neurodevelopmental disorders (NDDs). This review aims to provide a comprehensive overview of advancements in research pertaining to the microbiota-gut-brain axis in NDDs. Furthermore, we analyzed both the current state of research progress and discuss future perspectives in this field.

## Graphical Abstract

**Graphical Abstract:**
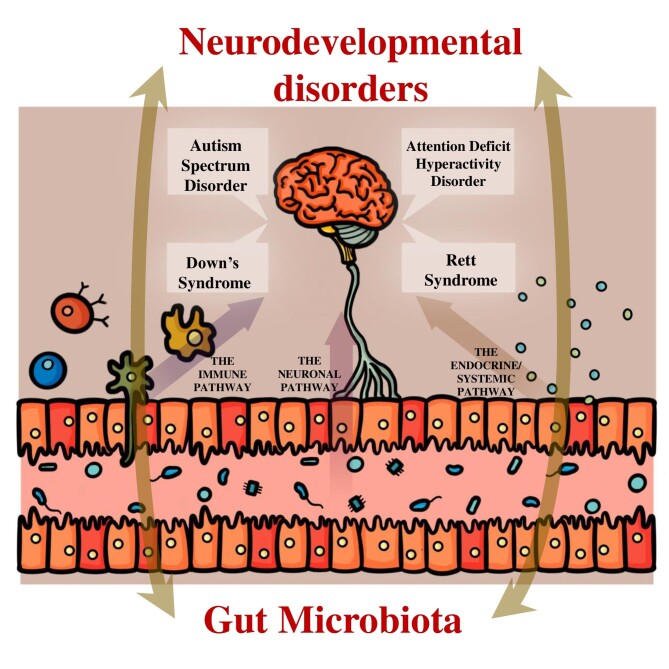

## Introduction

A staggering amount of research has found that the gut interacts with the brain in a bidirectional manner, known as the gut–brain axis. Inside the gut, the resident microorganism communities acting as a key regulator of the gut–brain axis have attracted even more attention. These communities include bacteria, fungi, viruses, and other forms of life, collectively known as the microbiome (Davenport et al., 2017). On the one hand, diverse physiological processes in the intestine, such as gastrointestinal (GI) motility, secretion, and digestive functions are modulated by the central nervous system (CNS) (Taché et al., 1980; Browning and Travagli, 2014). On the other hand, the gut microbiome influences brain function neurally, humorally, and immunologically (Dinan and Cryan, 2017; Maniscalco and Rinaman, 2018; Agustí et al., 2018). To be more specific, it is now widely accepted that this interaction is conducted through three major pathways, the immune pathway, the neuronal pathway, and the endocrine/systemic pathway, with interactions and crosstalks between these three (Agirman and Hsiao, 2021).

According to the diagnostic and statistical manual of mental disorders (DSM-5) (American Psychiatric Association, 2013), NDDs are a group of conditions which manifest during the developmental period and typically occurs in early development. NDDs are generally characterized by deficits in terms of personal, social, academic, and occupational functions. Typical NDDs include autism spectrum disorder (ASD), attention deficit hyperactivity disorder (ADHD), as well as certain types of learning and motor disabilities.

As researchers look deeper into the overlapped area between microbiology and neuroscience, it is becoming more apparent that the gut microbiota has a strong correlation with NDDs (Table 1). Numerous researchers have examined the physiological and pathophysiological mechanisms influenced by the gut microbiota in NDDs. This review aims to provide a comprehensive overview of advancements in research pertaining to the microbiota-gut-brain axis in NDDs. Furthermore, we will analyze both the current state of research progress and future perspectives in this field to provide a more thorough understanding of the topic.

**Table 1.**
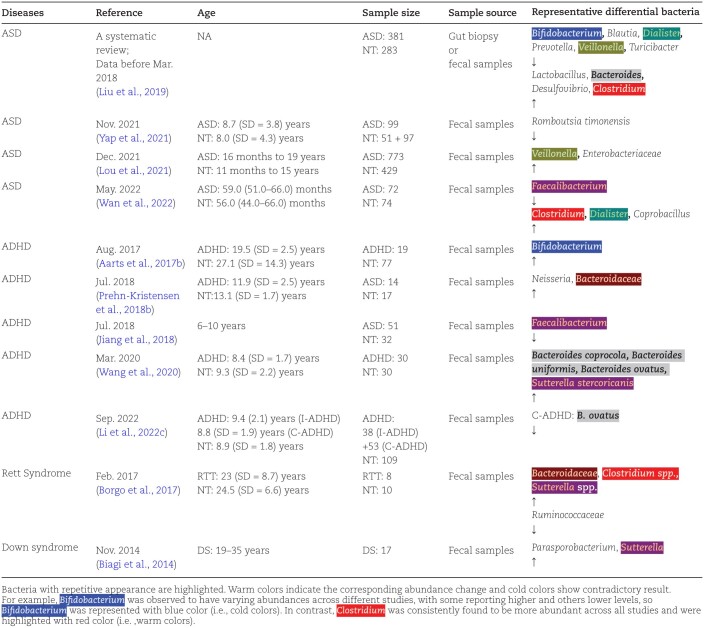
Summary of gut microbiota changes in NDDs.

## NDDs and the immune pathway mediated by gut microbiome

**Figure 1.**
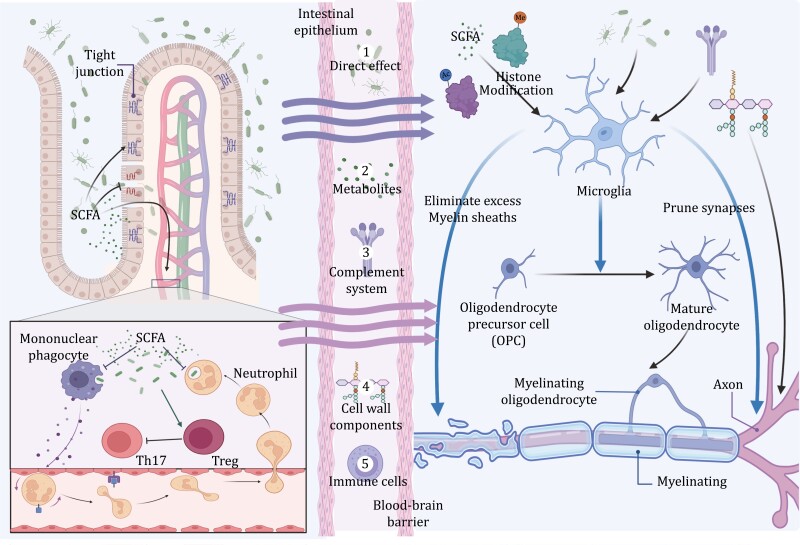
**The immune pathway.** Along the immune pathway, the gut microbiota regulates the brain by the bacteria themselves, microbial-derived metabolites (e.g., SCFAs, secondary bile acids, and amino acid metabolites), bacterial cell wall components (e.g., peptidoglycan, LPS), as well as microbial-intrigued immune cells and their secretory factors. The gut microbiota modulates enteric immunity in terms of the intestinal barrier, peripheral immune cells, and cytokines. For example, SCFAs derived by the gut microbiota seem to maintain a symbiotic relationship with the host by suppressing immune responses and protecting commensal bacteria from elimination, while also enhancing enteric barriers and reduce gut permeability to prevent invasion by harmful microorganisms. Proofs are that SCFAs have restrictive effects on neutrophil chemotaxis and mononuclear phagocyte system, promoting effect on regulatory T cells (Treg cells) and SCFAs can alleviate gut epithelium injury and regulate tight junctions. Meanwhile, in the CNS, microglia act as an important agent in neurodevelopment through their functions of synaptic pruning, neural progenitor cells (NPCs) pool supervision, neurogenesis regulation, etc. The gut microbiota affects microglia from different ways, exerting considerable effects on the neurodevelopmental process.

The CNS is vulnerable to various perturbations during development and altered immunological conditions may contribute to pathological processes in NDDs (Rodier, 1994; Han et al., 2021). Specifically, the gut microbiota interacts with the immune system by the residence itself, microbial-derived metabolites, such as short-chain fatty acids (SCFAs), secondary bile acids, and amino acid metabolites, and other bioactive molecules, such as microbe-associated molecular patterns (MAMPs) (Cryan et al., 2019). Together, they modulate local immunity within the gut, affecting the CNS through systemic circulation, and also play a modulating role with microglia as a mediator (Fig. 1).

Gut microbiota act as important regulators of the enteric immunity locally. Bacteria, along with bacterial-derived metabolites, are required to traverse the intestinal barrier to enter the circulation. Consequently, the intestinal barrier assumes a significant role in various physiological processes, as well as in the preservation of homeostasis within the CNS (Rothhammer et al. 2016; Fung *et al*. 2017; Pellegrini et al. 2018; Brescia and Rescigno 2021). For instance, one of the most explored microbial-derived metabolites, SCFAs, which are saturated fatty acids with fewer than six carbon atoms (Wong et al., 2006), have been found to alleviate gut epithelium injury (Chen *et al*., 2018; Li et al., 2022b) and regulate tight junctions (Zheng *et al*., 2017), therefore strengthening intestinal immunological barriers to reduce gut permeability and stopping pathogenic factors from invasion (Maslowski et al., 2009; Chang et al., 2014; Corrêa-Oliveira *et al*., 2016; Rodrigues *et al*., 2016). In contrast, dysbiosis of the gut microbiota may lead to alterations in the gut barrier, resulting in a “leaky gut” (Fasano, 2020) and making for the translocation of pathogens into the portal and systemic circulation, which contributes to neuroinflammation in CNS disorders such as ASD (Theoharides *et al*., 2013; Fasano, 2020). Apart from regulating the intestinal barrier, the gut microbiota also plays a role in the translocation of immune cells from the gut to the brain. In some cases, the term “translocation” stands for the process during which the gut microbiota interacts with immune cells locally and “trains” them to relocate to the CNS in order to perform certain functions. One example is that the gut microbiota activates a group of IFNγ^+^ NK cells that will in turn migrate to the CNS and induce the production of a type of anti-inflammatory astrocytes. These astrocytes induce cellular apoptosis in T cells through Tumor necrosis factor-related apoptosis-inducing ligand-decoy receptor 5 (TRAIL-DR5) signals and inhibit neuroinflammation as a result (Sanmarco et al., 2021). In addition to IFNγ^+^ NK cells, meningeal IgA^+^ plasma cells that are missing or decreased in germ-free (GF) mice are also confirmed by B-cell receptor sequencing to be originated from the gut. This type of plasma cells can migrate from the gut to the CNS, especially the meninges. Once into the meninges, these IgA^+^ plasma cells will prevent pathogenic factors from entering by trapping them in the dural sinuses, thus guarding the developing brain against infection, which highlights the role of the gut microbiota in preventing infectious encephalitis caused by pathogenic bacteria through training B-cell immunity (Fitzpatrick et al., 2020). Interestingly, in the case of T helper 17 cells (Th17 cells), translocation can produce inverse effects under certain circumstances. Th17 cells normally play a role in maintaining homeostasis in the gut and can exhibit proinflammatory effects when they migrate to the CNS, which is related to the tissue heterogeneity of these cells. In the experimental autoimmune encephalomyelitis (EAE) mouse models, there are both SLAMF6^+^ stem cell-like Th17 cells, which are modulated by the gut microbiota, that maintain homeostasis, and CXCR6 Th17 cells may migrate to the CNS to cause neuroinflammation (Schnell *et al*., 2021).

**Figure 2.**
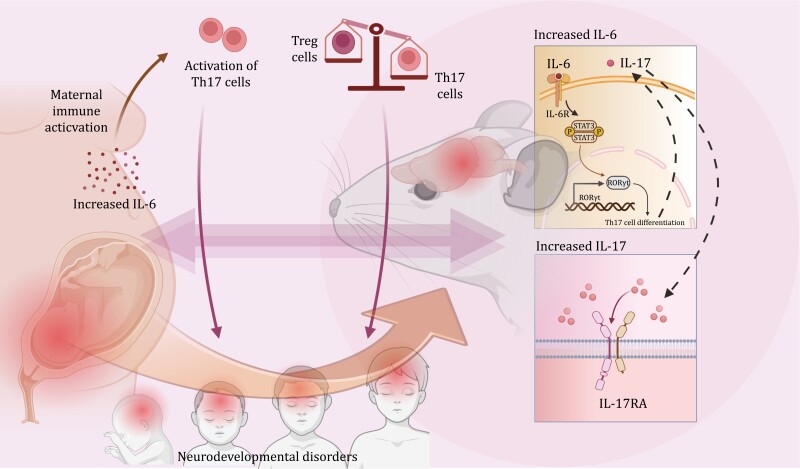
**Maternal immune activation.** MIA is associated with an increased risk of NDDs in the fetuses. One of the important cytokines for MIA is IL-17, which is produced mainly by Th17 cells. The gut microbiota is able to affect IL-17a levels and mucosal immunity as a whole by regulating the balance between Th17 cells and Treg cells. Besides, the production of IL-17 is also stimulated by elevated IL-6 levels during inflammation in pregnant females. Based on evidence from animal models, inside the CNS, IL17RA is located predominantly in the cortical neurons of S1DZ and elevated IL-17a levels lead to overactivation in these neurons, which then induces abnormalities in terms of social behaviors in MIA offsprings. Another important cytokine is IL-6. IL-6 not only affects the activation of Th17 cells, as is discussed above, but also affects neurons themselves by inducing transcriptional synaptogenesis through STAT3-dependent production of *RGS4*, which increase glutamatergic synapse density and disrupt hippocampal connectivity.

Meanwhile, the gut microbiota modulates neurodevelopment through its cell wall components as well as its regulation of cytokines via the systemic circulation. In terms of the circulating cell wall components, an important molecule is peptidoglycan. Peptidoglycan fragments are derived from bacterial cell walls and can cross the blood–brain barrier and activate pattern recognition receptors (PRRs) in the brain. These PRRs are widely expressed in the perinatal placenta and the brain at different neurodevelopmental stages (Gonzalez-Santana and Diaz Heijtz, 2020). For example, the recognition of peptidoglycan in the developing prefrontal cortex, the striatum, and the cerebellum may act to regulate synaptogenesis (Arentsen et al., 2017). Through the modulation of neurodevelopmental processes, peptidoglycan can further affect social behavior, anxiety, and stress responses, which play a role in autism spectrum disorder (Gonzalez-Santana and Diaz Heijtz, 2020). In addition to cell wall components, cytokines can also be found in the circulation and when reaching the CNS, may have diverse impacts on various neurodevelopmental processes, including neurogenesis, gliogenesis, and neuronal migration, etc. (Garlanda et al., 2013; Zengeler and Lukens, 2021). As such, the involvement of cytokines in NDDs proves a promising subject, and in recent years, this has been supported by studies on maternal immune activation (MIA). Various inflammatory factors in the maternal generation are associated with an increased risk of NDDs in the fetuses, which is possibly related to an underdeveloped blood–brain barrier (Han et al., 2021; Lu et al., 2023). Since retinoic acid receptor-related orphan nuclear receptor gamma t (RORγt)-dependent effector T lymphocytes are reported to be necessary for MIA-induced behavioral abnormalities (Choi et al., 2016), and Th17 cells not only fit into the RORγt-dependent effector T cell family but also experience a significant upregulation in MIA offspring, it can be inferred that interleukin-17 (IL-17) is one of the indispensable cytokines in MIA (Choi et al., 2016; Hoogenraad and Riol-Blanco, 2020). The gut microbiota is able to affect IL-17a levels and mucosal immunity as a whole by regulating the balance between Th17 cells and Treg cells (Pandiyan et al., 2019). For example, the administration of microbial-derived secondary bile acids, such as 3-oxoLCA and isoalloLCA, inhibits the differentiation of Th17 cells and promotes the differentiation of Treg cells by binding to RORγt and inducing the production of reactive oxygen species (ROS), respectively (Hang et al., 2019). SCFAs are another metabolite that has also been found to regulate Th17 and Treg cells. In the case of SCFAs, this is mainly achieved through histone deacetylase (HDAC) inhibition and G-protein coupled receptor (GPR) activation (Dalile et al., 2019). Interestingly, an overproduction of SCFAs increases Th17 cells and leads to the dysfunction of Treg cells during inflammation, suggesting that their functions could be paradoxical or concentration-dependent (Pandiyan et al., 2019). Aside from the microbial regulation of the balance between Treg and Th17 cells, the production of IL-17a is also stimulated by the other interleukins. For example, the secretion of IL-6 by the dendritic cells in the small intestine has been found to promote IL-17a production in pregnant females during inflammation, which indicates its role in MIA (Kim et al., 2017). Based on evidence from animal models, interleukin-17 receptor A (IL17RA) has been detected predominantly in the primary somatosensory cortex dysgranular zone(S1DZ), and elevated IL-17a levels lead to overactivation in the neurons of this area, which then induces abnormalities in terms of social behaviors in MIA offspring (Hoogenraad and Riol-Blanco, 2020). However, S1DZ is just a tiny part of a potentially extensive network that regulates MIA-related behavior, and more brain regions remain to be further appreciated (Shin Yim et al., 2017). Another cytokine that plays a role in MIA is interleukin-6 (IL-6). As mentioned above, IL-6 may affect the activation of Th17 cells and in turn, impact IL-17 concentrations. Besides this indirect function, IL-6 also acts directly on neurons to induce transcriptional synaptogenesis through *STAT3*-dependent production of the regulator of G protein signaling 4 gene (*RGS4*) downstream, and increased prenatal IL-6 levels promote the density of glutamatergic synapses and disrupt hippocampal connectivity (Mirabella et al., 2021). Still, it should also be noted that the correlation between abnormalities in the gut microbiota, cytokines, and neurodevelopment makes it possible to target NDDs by managing microbial compositions. In a recent study, the supplementation of *Lactobacillus reuteri* is found to improve the β-diversity of the gut microbiota in the offsprings and promote the metabolic functions of their brain (Lu et al., 2023) (Fig. 2).

Aside from regulating local immune players to impact the CNS indirectly and modulating the developing brain systemically, the gut microbiota also affects neurodevelopment with microglia as the mediator. This deserves our attention because microglia have been identified to be closely involved in neurodevelopment, and abnormalities in their morphology, as well as functions, may play a role in NDDs [reviewed by (Zengeler and Lukens, 2021)]. As the professional phagocytes of the brain, these cells serve the function of clearing debris during the proliferation of nerve cells as well as engulfing various live cells, known as “phagoptosis” (Harry, 2013; Brown and Neher, 2014). In the developing CNS, microglia perform the critical function of monitoring the pool of neural progenitor cells (NPCs) and regulating the process of neurogenesis. This regulatory role is achieved through the engulfment of oligodendrocyte progenitor cells (OPCs) and the modulation of neural precursor cell size in the cerebral cortex (Cunningham et al., 2013; Nemes-Baran et al., 2020). Additionally, microglia selectively eliminate excess myelin sheaths, thereby modifying myelination during neurodevelopment (Hughes and Appel, 2020). Microglia can also prune synapses, and once this ability is compromised, there will be an excess of dendritic spines and immature synapses, which may lead to immature brain circuitry (Paolicelli et al., 2011). This process possibly occurs through the complement system, especially the complement component 3 (C3) and complement component 3 receptor (CR3) signaling, since mice without CR3, C3, and the C-X3-C motif chemokine receptor 1 gene (*CX3CR1*) undergo a decrease in microglial synaptic pruning in the developing visual system as well as the hippocampus (Paolicelli et al., 2011; Schafer et al., 2012). The gut microbiota plays a part in diverse events of the microglia, including their maturation and aging, as well as their functions, and microbial-derived metabolites promote microglia restoration in GF mice (Erny et al., 2021; Mossad et al., 2022). Indeed, an intact microbiota is not only indispensable for the localization of forebrain microglia during neurodevelopment but is also important in modulating the microglial expression of the complement signaling pathway and the synaptic remodeling factor complement C1q. This allows it to restrain neurite complexity and regulate forebrain neurons to have a promotive function in social behaviors (Bruckner et al., 2022). Meanwhile, GF mice are found to exhibit a different landscape of genes that are related to the complement system (Matcovitch-Natan et al., 2016), since microbial-derived metabolites such as SCFAs, have been found to regulate the C3 signaling within the CNS and may play a role in the C3/CR3-dependent microglial elimination of synapses (Lai et al., 2021). Other than SCFAs, aryl hydrocarbon receptor (AHR) agonists such as indole, indoxyl 3-sulfate, indole-3-propionic acid, and indole-3-aldehyde (Rothhammer et al., 2016), which are metabolized from dietary tryptophan by the gut microbiota (Quintana and Sherr, 2013), also modulate the production of transforming growth factor α (TGFα) and vascular endothelial growth factor B (VEGF-B) by the microglia, controlling the inflammation in the CNS as a consequence (Rothhammer et al., 2018).

## The endocrine/systemic pathway in neurodevelopment regulated by gut microbiome

**Figure 3.**
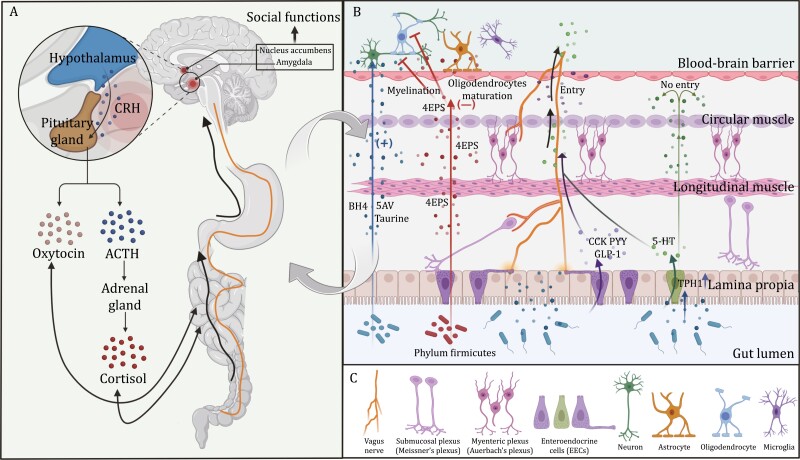
**The endocrine (systemic) pathway and the neuronal pathway.** (A) The HN axis and the HPA axis play an important role in the endocrine (systemic) pathway. The neuropeptide oxytocin acts as an important component of the HN axis, modulates the interplay within the serotonergic system in the nucleus accumbens and the marginal activity in the amygdala, thus regulating social functions. Along the HPA axis, peripheral cortisol levels have been found to be significantly higher in patients with ASD and bacterial species, such as *Enterococcus faecalis*, inhibit the elevated glucocorticoid levels after social stress and promote social behaviors in mice, indicating that the gut microbiota is capable of influencing HPA axis. (B) The gut microbiota can produce neuroactive molecules (e.g., 5AV, taurine, and 4EPS) directly and these molecules have diverse effects on neurodevelopment processes (i.e., myelination, oligodendrocytes maturation). The gut microbiota also regulates the production of 5-HT in ECCs. However, metabolites like 5-HT cannot across the blood–brain barrier without the vagus nerve. Microbial-derived metabolites and other substances not only interact with the vagus nerve but also impact the ENS and the intestinal mechanosensory. The vagus nerve is an important agent between the endocrine (systemic) pathway and the neuronal pathway.

The gut microbiota releases diverse metabolites and produces neurotransmitters and neuromodulators (Cussotto et al., 2018; Agirman and Hsiao, 2021), both of which can modulate neurodevelopment. These metabolites or products include gamma-aminobutyric acid (GABA), serotonin, dopamine, norepinephrine, acetylcholine, histamine, secondary bile acid, 4-ethylphenyl sulfate (4EPS), SCFAs, and so on (Agirman and Hsiao, 2021; Lh et al., 2021; Needham et al., 2022). Increasing evidence showed that bacterial-derived metabolites can promote neurodevelopment; however, some bacteria may also play a causal role in NDDs. An example of microbial-derived metabolites with the former function would be taurine, which is a neuroactive amino acid metabolite of the gut microbiota that is deficient in the dams of ASD mouse models. Indeed, the oral administration of taurine to the ASD dams during pregnancy has been found to reduce repetitive behaviors in their offspring. Similarly, 5-aminovaleric acid (5AV), another amino acid metabolite of the gut microbiota, also leads to a decrease in repetitive behaviors and an improvement in social interactions. This indicates their positive role in neurodevelopment (Sharon et al., 2019). Additionally, it has been found that tetrahydrobiopterin (BH4), a metabolite induced by *L. reuteri*, can improve social deficits in mouse models of ASD, which may be explained by a BH4-induced promotion in synaptic transmission mediated by the social reward mechanism in the brain (Buffington et al., 2021). In contrast, 4-ethylphenyl (sulfate), or 4EP(S), which is produced by certain members of the Firmicutes phylum, such as *Bacteroides ovatus*, has emerged to be negatively related to neurodevelopment (Hsiao et al., 2013; Lh et al., 2021; Needham et al., 2022) since elevated levels of 4EP(S) are detected in ASD patients and the *CNTNAP2* (contactin associated protein 2) mouse models of ASD (Needham *et al*., 2021). A positive correlation has been found between 4EP(S) and repetitive behaviors whereas a negative correlation exists between this metabolite and communication, which suggests that 4EP(S) plays a role in the demonstrated manifestations of ASD (Hsiao *et al*., 2013). Mechanisms have been elucidated by some studies that, once inside the CNS, 4EP(S) can inversely impact myelination, an important process in the development of the brain, and thus will influence ASD-related behaviors as a consequence (Berer *et al*., 2011; Hoban et al., 2016; Bonnefil et al., 2019; Pan et al., 2020) (Fig. 3B).

Apart from producing the above metabolites that are able to regulate neurodevelopment as well as other neurotransmitters and neuromodulators themselves, the gut microbiota also interacts with the enteroendocrine system to regulate neurodevelopment. The enteroendocrine system is composed of various enteroendocrine cells (EECs) which are capable of producing glucagon-like peptide 1 (GLP-1), peptide YY (PYY), cholecystokinin (CCK), substance P, and 5-HT (Gribble and Reimann, 2016). EECs can sense various microbial signals and also synapse with the vagal neurons to communicate with the CNS (Gribble and Reimann, 2016; Bellono et al., 2017). Specifically, certain bacteria strains act through deoxycholic acid, SCFAs, as well as other metabolites, to upregulate the levels of the rate-limiting enzyme in 5-HT biosynthesis, tryptophan hydroxylase 1 (TPH1), which can increase 5-HT levels in a subtype of EECs called the enterochromaffin cells (ECCs) (Alemi et al., 2013; Reigstad et al., 2015; Yano *et al*., 2015). Besides, LPS has also been found to play a part through toll-like receptor 4 (TLR4) despite that supporting evidence is limited (kidd et al., 2009). 5-HT concentration is suggested to affect social behaviors in ASD, which implies its potential role in the modulation of NDDs (de Theije et al., 2014). However, peripheral 5-HT is not able to pass through the blood–brain barrier (Donovan and Tecott, 2013). In recent years, an interconnected communication system between the gut microbiota, ECCs, and the vagus nerve has been discovered to explain the impact of peripheral 5-HT on neurodevelopment (Margolis *et al*., 2021). 5-HT3 and 5-HT4 receptors have been found on the vagus nerve, indicating that 5-HT may indirectly impact the CNS by acting through the vagus nerve (Bonaz *et al*., 2018; Bhattarai *et al*., 2018). Meanwhile, emerging studies have provided new clues to the direct connection between the EECs and the vagus nerve, suggesting that there is a specific cell type named the neuropods which form synapses with the vagus nerve. This allows for the fast transmission of signals from the gut to the brain (Kaelberer *et al*., 2020).

In terms of neuroendocrine signaling, hormones such as oxytocin, vasopressin, and glucocorticoid have been reported to be regulated by the gut microbiota and, in turn, affect neurodevelopment (Cussotto *et al*., 2018; Lh *et al*., 2021). It should also be noted that the gut microbiota can decompose and produce hormones as well. For example, *Klebsiella aerogenes* are shown to degrade estradiol because of the expression of the 3β-HSD (3β-hydroxysteroid dehydrogenase) gene(Li *et al*., 2023). Two of the related axes in this process are the hypothalamic–neurohypophyseal (HN) axis and the hypothalamic–pituitary–adrenal (HPA) axis (Dayanithi *et al*., 1987; Stephens and Wand, 2012). First, as an important component of the HN axis, the neuropeptide oxytocin modulates the interplay within the serotonergic system in the nucleus accumbens and the marginal activity in the amygdala, thus regulating attachment, aggression, social fear, social learning, as well as other complex social functions (Heinrichs *et al*., 2009; Dölen *et al*., 2013; Neumann and Slattery, 2016; Fineberg and Ross, 2017). A critical etiological factor in social defects related to the crosstalk between the gut microbiota and oxytocin is the maternal high-fat diet (MHFD) since in MHDF offspring, there is a decrease in both synaptic improvements of the ventral tegmental area (VTA) during social interaction and the number of oxytocin immunoreactive neurons in the hypothalamus (Buffington *et al*., 2016). In mouse models, the administration of *L. reuteri*, a bacterial strain that can be downregulated by MHFD, reserves oxytocin decrease, synaptic deficits, and the plasticity of VTA, thereby improving social behaviors and consequently relieving ASD (Buffington *et al*., 2016; Francis and Dominguez-Bello, 2019). This function is possibly associated with the afferent vagus nerve as well because *L. reuteri-*derived metabolites can act on the vagus nerve and monitor the oxytocin–dopamine reward system in the brain (Sgritta *et al*., 2019a). Second, along the HPA axis, peripheral cortisol levels have been found to be significantly higher in patients with ASD, indicating that the HPA axis plays a role in ASD (Gao *et al*., 2022a). In 2021, Wu *et al*. identified a decrease in social activities and an increase in neuronal activity after social stress that are both related to abnormal corticosterone levels, suggesting that the crosstalk between the gut microbiota and the HPA axis may play a role in social behaviors (Wu *et al*., 2021). Bacterial species, such as *Enterococcus faecalis*, inhibit the elevated glucocorticoid levels after social stress and promote social behaviors in mice (Wu *et al*., 2021). Altered mRNA expression can be detected in both NMDA and 5-HT1A receptors in GF mice, suggesting that this is related to the regulation of the gut microbiota on the expressions of the receptors. Once NMDA and 5-HT1A receptors are regulated, corticotropin-releasing factors (CRFs) released from the hypothalamus will be affected as a result (Neufeld *et al*., 2011) (Fig. 3A).

## Neuronal pathways of bidirectional gut–brain communications

Neuronal pathways of bidirectional gut–brain communications are intuitive. Unique among visceral organs, the GI tract has its own intrinsic nervous system—the enteric nervous system (ENS) (Uesaka *et al*., 2016; Marklund, 2022). The extrinsic nervous system plays a significant role in GI physiology, among which the vagus nerve has been a key focus in recent research. The vagus nerve is the tenth cranial nerve, of which the hepatic and celiac branches innervate the gut. Sensory/afferent and motor/efferent fibers are intermingled in the vagus nerve. The sensory fibers originate from neurons of the nodose ganglion, whereas the motor fibers come from neurons of the dorsal motor nucleus of the vagus (DMV) and the nucleus ambiguous. Vagal afferents treat chemical signals as important gut inputs. As previously mentioned, EECs release neurohormones including CCK, GLP-1, PYY, serotonin, etc. (Chambers *et al*., 2013). These hormones spread to adjacent afferent terminals and bind vagal sensory neurons’ receptors. Such chemoreceptors include CCKAR (for CCK), GLP1R (for GLP-1), and HTR3A (for serotonin) (Williams *et al*., 2016). CCK (Li *et al*., 2022a), GLP-1 (Müller *et al*., 2019; Borgmann *et al*., 2021), and PYY (Steinert *et al*., 2017) function mainly as the regulators of food intake and digestion either directly on the brain through the circulatory system, or indirectly via the vagal-brain pathway. In addition, CCK can cause some direct central nervous responses. By practicing the bilateral injection into the nodose ganglia of CCK-SAP to decrease CCKAR expression, a study proves that such CCK block up causes GI vagal afferents inhibition and results in attenuated anxiogenic effects of refeeding (Krieger *et al*., 2022). Importantly, by performing vagotomy, studies have found peptides or neurotransmitters like serotonin, oxytocin (Sgritta *et al*., 2019b), GABA (Bravo *et al*., 2011a), and brain-derived neurotrophic factor (BDNF) (Bercik *et al*., 2011) which are derived from microbes-derived metabolites or microbe-triggered host secretion are not able to have the supposed effect on the brain without the vagus nerve. For instance, *Lactobacillus rhamnosus* (JB-1) has a direct effect on neurotransmitter receptors that it can induce region-dependent alterations in GABAB1b mRNA in the brain. The process is mediated by the vagus nerve because such rescue cannot be achieved in mice operated with a bilateral subdiaphragmatic vagotomy (Fig. 3B).

Apart from the molecules mentioned above, some immune factors produced by the stimuli of the microbiome may also have the capability of activating the vagal afferents to prepare for the immune response. For example, in an early study, gut inoculation of *Campylobacter jejuni* in mice has been reported to result in direct activation of the vagal sensory ganglia and the nucleus tractus solitarius (NTS) in the medulla oblongata (Goehler *et al*., 2005). *Campylobacter jejuni* is the leading cause of bacterial diarrhoeal disease in many areas of the world (Burnham and Hendrixson, 2018; Malik *et al*., 2022) and its administration has been reported to induce anxiety-related behaviors (Lyte et al., 2002). *Campylobacter jejuni* is also causally linked with the development of the autoimmune peripheral neuropathy Guillain Barré Syndrome (GBS) (Malik *et al*., 2022). Such responses caused by *C*. *jejuni* may be mediated by the vagal afferents. It is noteworthy to mention that vagus nerve stimulation (VNS) may potentially serve as a therapeutic intervention for NDDs owing to its potent anti-inflammatory effects. Apart from the vagus nerve, the ENS, also acts as an essential mediator along the microbiota–gut–brain axis. Both animal experiments and cross-sectional studies have indicated that the ENS is involved in CNS disorders, especially in ASD, where GI comorbidities are frequently present (Rao and Gershon, 2016). Current evidence suggests that microbes-derived metabolites are capable of regulating enteric neuron functions, such as the excitability of enteric nerve endings, affecting the endocrine as well as immune pathways in an indirect manner and consequently interacting with the CNS (Vickers, 2017; Agirman and Hsiao, 2021). This type of overlap and crosstalk is also discussed in other sections of the review.

The GI tract is insensitive to cutting, crushing, or burning (Gray *et al*., 2021). However, mechanical signals (i.e., distension, contraction, flow (shear)) are significant to the gut since nearly all gut functions require the sense of forces emanating from the digestion of intraluminal contents and organ activity (Mercado-Perez and Beyder, 2022). In addition, organoid evidence continues to emerge in support of the important role mechanical signals have played in the morphogenesis of the GI tract (Poling *et al*., 2018; Yavitt *et al*., 2023). The term “mechanosensor” refers to mechanical signal receivers, proteins that convert mechanical stimulus into an intracellular electrochemical signal. The landscape of molecular mechanosensors of the vagus nerve of the GI tract is currently unclear. Nevertheless, two comprehensive reviews covering this topic have given the readers an overview of mechanosensing in the GI tract (Kim *et al*., 2022; Mercado-Perez and Beyder, 2022). Mechanosensors sense mechanical stimulus by different ion channels (such as TRP, ASICs, PIEZO, etc.), and signals are transported to the brain partly by the vagus nerve, and eventually different mechanosensory circuits form. Other than chemical signals and mechanical signals, there are some other signals vagal afferents detect from the gut. In 2015, Bohórquez *et al*. discovered a direct connection between a special subset of EECs and vagal neurons by making synapses (Bohórquez *et al*., 2015). Such EECs are named neuropods and following research found that they use glutamate as a neurotransmitter to transmit excitatory signals to vagal neurons. Glutamate is a fast-passing neurotransmitter, while neuropeptides such as CCK are slow-passing neurotransmitters (paracrine communication) (Kaelberer *et al*., 2018). The formed synapses enable ultrafast millisecond transmission from EECs to vagal sensory neurons. Further studies of neuropods have found out that neuropods resemble sensory cells of the nervous system. They influence the preference for sugar over sweeteners by sensing the difference between these two and releasing different neurotransmitters to different cells in the vagus nerve (Buchanan *et al*., 2022). Another new vagal sensory modality has been identified that sensory neurons can detect visceral osmolality changes and translate them into hormonal signals to regulate thirst circuit activity through the HPA pathway (Ichiki *et al*., 2022). Notably, as can be inferred from above, the gut microbiota mainly interacts with the mechanosensors indirectly to regulate neurodevelopment, but direct evidence remains to be explored (Fig. 3B).

## The gut microbiota and NDDs

ASD is an early-onset NDD that is defined by impaired social interaction, deficits in communication, and the presence of repetitive, stereotyped behaviors (American Psychiatric Association, 2013). The etiology of ASD is a hot issue that has not been completely elucidated while two main factors are regarded as the attributions for ASD—the genetic and the environmental factors. Some large-scale sequencing studies have identified more than a hundred ASD highly relatable genes and SNVs/indels, SVs, tandem repeats, etc. (Satterstrom *et al*., 2020; Willsey *et al*., 2022; Trost *et al*., 2022; Yuan *et al*., 2023). Environmental factors include pathogen exposure, nutritional deficiencies or overload, toxic exposure, allergies, etc. (Hisle-Gorman *et al*., 2018). As mentioned above, MIA is a representative example of how environmental factors increase the risk of developing ASD. Individuals with ASD often experience co-occurring GI symptoms, including constipation, diarrhea, and abdominal pain (Jolanta Wasilewska and Klukowski, 2015), and patients with GI symptoms are inclined to be more irritable, withdrawn, or hyperactive (Leader *et al*., 2022). The existence of such GI symptoms has raised interest in studying the possible influence of gut microbiota on the pathogenesis of autism. A number of studies report the significant difference in the gut microbiome composition between patients with ASD and healthy controls (see Table 1). As can be observed in several studies, there is an increase in the abundance of the *Clostridioides* genus (Liu *et al*., 2019; Wan *et al*., 2022). In fact, the *Clostridioides* genus is one of the most frequently detected dysregulated bacteria in patients with ASD despite unpreventable bias from the heterogeneity (Zheng *et al*., 2021). Interestingly, two specific metabolites derived from microbiota, especially from the *Clostridioides* genus, have been well studied for their effects on microglial cells and processes like neuroinflammation and microglial phagocytosis. One is 4-EP(S), which has been previously reviewed at the endocrine/systemic pathway section and the other is p-Cresol sulfate (pCS) which originates from bacterially produced p-Cresol. Both pCS and 4EPS are products of microbial degradation of aromatic amino acids (AAAs) and some other AAAs and their intermediate or final products such as phenylalanine, tryptophan, and tyrosine are also important metabolites in ASD studies. The most classical example is serotonin, of which tryptophan is its precursor. Since the 1970s, it has been widely reported that inside autistic patients' blood, the concentration of serotonin is abnormally high (Hanley, 1977) while contradictorily, the concentration inside the brain is rather low (Chugani *et al*., 1999). Nevertheless, the specific mechanism behind this phenotype has not been explained clearly and the application of selective serotonin reuptake inhibitors (SSRIs) has not been proven effective (Fattorusso *et al*., 2019). Recently, the metabolism of another product of tryptophan—kynurenine, has been reported as abnormal in ASD murine models (Lavelle and Sokol, 2020). Brain samples obtained from the frontal cortex showed higher concentrations of 3-hydroxy kynurenine and 3-hydroxy anthranilic acid which are neurotoxic and often converted into quinolinic acid in reactive microglial cells and therefore very likely to cause neuroinflammatory outcomes (Parrott *et al*., 2016; Murakami *et al*., 2019). Additionally, an 'inflammation hypothesis' was raised to explain the GI symptoms in autistic children. There is increasing evidence that GI symptoms in autistic children may be due to the inflammatory state in the gut and the microbiota has played a potential role in promoting this process (Puricelli *et al*., 2022). Autistic children are more likely to have a 'leaky gut' and owing to this higher permeability, the CNS is highly exposed to proinflammatory cytokines (Ashwood *et al*., 2011). As stated above, under the dominance of genetic factors, the investigation of the mechanistic relationship between the gut microbiota, as an environmental factor within the host, and the host in regulating the occurrence and development of ASD, represents a highly worthy scientific inquiry. In 2019, our research team demonstrated in *Drosophila melanogaster* that mutations in autism-associated gene KDM5 can alter intestinal immunity, thereby affecting the behavior of the fruit fly through the influence of the gut microbiota (Chen *et al.*, 2019). Similarly, as a recent publication in ASD omics research suggested, long-chain polyunsaturated fatty acids may causally contribute to sleep disturbances mediated by the FADS gene cluster and with potential mediation by the microbiota, sleep disturbances and unhealthy diet have a convergent lipidome profile (Yap *et al*., 2023).

Various treatments that target the microbiota–gut–brain axis have been put forward in recent years, with the aim of restoring the balance of the gut microbiota. Probiotics are one of the most explored therapeutic methods and have already produced positive outcomes in NDDs (George Kerry *et al*., 2018). Several studies addressed the effects of the administration of probiotics in ASD. For example, in ASD murine models, the supplementation of the probiotic *L*. *reuteri* proved effective in improving social behaviors, although clinical evidence remains limited (Kong *et al*., 2020). Another commensal bacterium, *Bacteroides fragilis*, is also believed to act as a probiotic and can fix the permeability of the gut and, in turn, alleviate ASD symptoms (Gilbert *et al*., 2013). Furthermore, a recent study revealed that *Chd8*^*+/*−^ mouse models of ASD exhibit elevated serum glutamine levels due to a high expression of amino acid transporters in the intestine as well as increased glutamine levels in the brain, which is associated with the manifestation of ASD symptoms. The supplementation of *Bifidobacterium longum* has been found to downregulate intestinal amino acid transporter expression and thus ameliorate ASD-like behaviors in mouse models, which also demonstrates its therapeutic potential in ASD (Yu *et al*., 2022). Apart from probiotics, prebiotics is also under exploration. Accompanied by the exclusion diet, a 6-week administration of the prebiotic, Bimuno® galactooligosaccharide (B-GOS®) in 30 autistic children showed positive results, indicating that the supplementation of prebiotics is also potentially beneficial (Grimaldi *et al*., 2018). Additionally, studies on fecal microbiota transplantation (FMT), or microbiota transfer therapy (MTT), have also identified its efficacy in ASD treatment. A clinical trial that followed up 18 ASD participants found that FMT restored bacterial diversity and richness in both *Bifidobacteria* and *Prevotella*. Besides, an improvement in both GI symptoms as well as the core symptoms of ASD that were maintained throughout 2 years of time was also observed (Kang *et al*., 2019). In favor of this result, in 2021, Li *et al*. also observed a long-lasting benefit in GI and behavioral symptoms, which was associated with *Eubacterium coprostanoligene* (Li *et al*., 2021). It should be noted that the effect of FMT is not only affected by the gut microbiota composition of the donor but also that of the recipient. Other factors, such as the methods of administration and the choice of preservation of the FMT sample, also play a role in the effects of FMT (Ng *et al*., 2020). Therefore, FMT is not a one-size-fits-all solution in the treatment of ASD.

Notably, Centers for Disease Control and Prevention estimates that about 26% of people with ASD suffer from depression, and people with ASD are three times more likely to suffer from depression than the general population. Moreover, there are also considerable studies emphasizing the role of the gut microbiota in depression (Chang *et al*., 2022; Liu *et al*., 2023). Therefore, potential relations and mechanism between depression and ASD, are worth further studying.

ADHD is another commonly occurred NDD with an estimated prevalence of ~5% worldwide (Sayal *et al*., 2018). As shown in Table 1, alterations in the composition of the gut microbiota are found to be presented in people with ADHD. For example, decreased abundance of actinobacteria reduces the ADHD-RS-IV scores, and an increase in the genus *Bifidobacterium* and the family *Bacteroidaceae* has also been observed in adolescents with ADHD (Aarts *et al*., 2017a; Prehn-Kristensen *et al*., 2018a; Stevens *et al*., 2019). Additionally, low *B. ovatus* is also associated with cognitive deficits in ADHD (Li *et al*.). In terms of alpha and beta diversity, however, the results have been contradictory possibly due to the differences in the methodologies between studies. Meanwhile, the transplantation of the gut microbiota from people with ADHD generates ADHD-like behaviors in GF C57BL/6JOlaHsd mice, suggesting that altered gut microbiota composition plays a role in ADHD pathogenesis (Tengeler *et al*., 2020). Indeed, an altered microbiota–gut–brain axis contributes to the presence of the core symptoms of ADHD as well as the comorbidities, such as sleep disorders [reviewed by (Checa-Ros *et al*., 2021)]. In terms of treatment, probiotics, prebiotics, and synbiotics have all been demonstrated to be beneficial in the therapeutic interventions of ADHD, both directly and indirectly [reviewed by (Kalenik *et al*., 2021)]. For one thing, a double-blind randomized controlled trial has revealed that the application of synbiotics, which are a combination of pre-and probiotics, has been found effective in enhancing the emotional regulation of ADHD adults (Skott *et al*., 2020). For another, there have been accumulating studies proving that *L. rhamnosus* might improve the stability of the intestinal barrier locally and regulate GABA and GABA receptors in the CNS through the vagus nerve in the meantime. Both of them have an alleviating effect on ADHD development and symptoms (Isolauri *et al*., 2008; Enticott *et al*., 2010; Bravo *et al*., 2011b; Pärtty *et al*., 2015). In addition, diet also proves a possible solution. For instance, once metabolized by the gut microbiota, omega-3 (n-3) polyunsaturated fatty acids (PUFAs) will reduce ADHD-like behaviors by acting through the reinforcement-insensitive mechanism (Dervola *et al*., 2012). Along the immune pathway, omega-3 PUFAs also inhibit the activation of the NOD-, LRR-, and pyrin domain-containing protein 3 (NLRP3) inflammasome, which further decreases the secretion of IL-1β (Yan *et al*., 2013). In a meta-analysis, the administration of PUFAs is found to be favorable in ameliorating ADHD symptoms as well, although the credibility is not strong enough (Gao *et al*., 2022b). Still, PUFA supplementation is potentially promising in alleviating ADHD.

Besides ASD and ADHD, Rett Syndrome (RTT) is also a severe NDD. RTT is observed in girls and is characterized by progressive mental decline, motor dysfunction, and ASD-like behaviors. Dysbiosis in the gut has been observed in patients with RTT, suggesting that the gut microbiota plays a role in this NDD (Neier *et al*., 2021). Specifically, reduced richness in certain microbial taxa in *Bifidobacterium*, *Anaerostipes*, *Clostridium* XIVa, *Clostridium* XIVb, *Erysipelotrichaceae*, *Actinomyces*, *Lactobacillus*, *Enterococcus*, as well as *Eggerthella*, has been identified in RTT patients (Strati *et al*., 2016), while an enrichment was found in *Bacteroidaceae*, *Clostridium* spp., and *Sutterella* spp. (Borghi *et al*., 2017). It should be noted that an inflammatory profile is seen in female mouse models of RTT, suggesting that the immune pathway is possibly involved (Neier *et al*., 2021). Indeed, microglia have been found to play a part since activated microglia and loss of microglia through apoptosis are associated with the development of the condition (Lukens and Eyo, 2022). On the therapeutic scale, both prebiotics and probiotics are believed to have the potential for GI as well as behavioral dysfunctions in RTT, although specific solutions remain to be further appreciated (Borghi *et al*., 2017).

## Conclusion and future perspectives

Currently, the application of GF models, antibiotics, FMT, brain imaging, microbiome sequencing, and bioinformatics has brought us closer to understanding the microbiota–gut–brain axis [reviewed by (Cryan *et al*., 2019)]. Evidence from both preclinical and clinical research has indicated that the gut microbiota modulates diverse processes in the CNS and neurodevelopment, among which data accumulated primarily from preclinical studies have shown that the gut microbiota acts through the aforementioned three pathways along the microbiota–gut–brian axis to impact blood−brain barrier permeability, synaptic pruning, neurogenesis, neuronal signaling, and behaviors or emotions such as sociability, sensory, memory, learning, and stress (Erny *et al*., 2017; Vuong *et al*., 2017; Pronovost and Hsiao, 2019; Morais *et al*., 2021). However, various questions remain unsolved, and in some cases, information from studies may be conflicting.

Firstly, studies so far have established that there are three pathways along the microbiota–gut–brain axis, the endocrine/systemic pathway, the immune pathway, and the neuronal pathway, and interactions as well as overlaps between pathways have also been studied. However, there are potentially other novel components that might be involved in these pathways, such as autophagy and the endocannabinoid system, that are being increasingly recognized [reviewed by (Shoubridge *et al*., 2022)]. The roles and categorization of these components require further exploration. Despite our growing understanding, the exact mechanism of how various physiological processes of the CNS are affected by the gut microbiota remains to be addressed more elaborately as well.

Secondly, in spite of the accumulating evidence on the impacts of the gut microbiota on the differentiation and maturation of immune cells, studies on certain enteric immune cells remain immature. For instance, there have been limited studies on the microbial impacts on mast cells. Besides, it has long been established that the local immune systems are important in other organs such as the lung and the liver. Indeed, microbial dysbiosis of the lung has been found to impact the immunity of the lung, which plays a role in chronic lung diseases (O’Dwyer *et al*., 2016). However, the way that the gut microbiota regulates the immune systems in these organs to affect neurodevelopmental processes still needs to be further explored.

In addition, there have been data supporting both the genomic and non-genomic influence of the gut microbiota, especially the interplay of gut microbiota-derived metabolites and epigenetics involved in diverse cellular processes (Woo and Alenghat, 2022). As mentioned above, the most studied metabolites are SCFAs, with various studies on their effects on epigenetics as well as cellular receptors and intracellular signaling cascades (Dalile *et al*., 2019). However, there is still a paucity of data on the exact mechanisms of how various other microbial-derived metabolites, and microbial cell wall components affect neurodevelopmental processes through the immune, neuronal, and endocrine pathways via epigenetics regulations.

Meanwhile, in recent years, optogenetic technology has emerged as a promising assistance in neuroscience research. With the help of this technology, the crosstalks between the heart and the brain have gradually been revealed (Veerakumar *et al*., 2022; Hsueh *et al*., 2023). However, the application of this technology in understanding the crosstalks between the gut and the brain in NDDs remains in its infancy. Optogenetic technology has been found to be able to control the metabolism of the gut microbiota and regulate engineering bacteria that are taken in for therapeutic purposes, demonstrating its potential in neuroscience research (Hartsough *et al*., 2020). Therefore, more studies need to be carried out to make full use of optogenetic technology to further appreciate the impacts of the gut microbiota on neural circuits.

Finally, as discussed in the previous reseaches, therapeutic interventions such as probiotics, prebiotics, synbiotics, diet, and FMT, have been accepted as promising in NDDs. However, although there have been a large amount of data coming from animal experiments, evidence from humans is still insufficient (Ng *et al*., 2020). Meanwhile, customized plans for different patients need to be standardized so that the effects of the aforementioned therapeutic methods can be brought to full play.

## Abbreviation

3β-HSD: 3β-hydroxysteroid dehydrogenase
4EPS: 4-ethylphenyl (sulfate)
5AV: 5-aminovaleric acid
5-HT: 5-hydroxytryptamine
AAAs: aromatic amino acids
ACTH: adrenocorticotropic hormone
ADHD: attention deficit hyperactivity disorder
AHR: aryl hydrocarbon receptor
ASD: autism spectrum disorder
BDNF: brain-derived neurotrophic factor
BH4: tetrahydrobiopterin
C3: complement component 3
CCK: cholecystokinin
CNS: central nervous system
*CNTNAP2*: contactin associated protein 2
CR3: complement component 3 receptor
CRFs: corticotropin-releasing factors
CRH: corticotropin-releasing hormone
*CX3CR1*: C-X3-C motif chemokine receptor 1 gene
DMV: dorsal motor nucleus of the vagus
DSM-5: diagnostic and statistical manual of mental disorders
EAE: experimental autoimmune encephalomyelitis
EECs: enteroendocrine cells
ENS: enteric nervous system
FMT: fecal microbiota transplantation
GABA: gamma-aminobutyric acid
GBS: Guillain Barré Syndrome
GF: germ-free
GLP-1: glucagon-like peptide 1
GPR: G-protein coupled receptor
HDAC: histone deacetylase
HN: hypothalamic–neurohypophyseal;
HPA: hypothalamic–pituitary–adrenal axis
IL-17: interleukin-17
IL17RA: interleukin-17 receptor A
IL-6: interleukin-6
MAMPs: microbe-associated molecular patterns
MHFD: maternal high-fat diet
MIA: maternal immune activation
MTT: microbiota transfer therapy
n-3: omega-3
NDDs: neurodevelopmental disorders
NLRP3: NOD-, LRR-, and pyrin domain-containing protein 3
NPCs: neural progenitor cell
NTS: nucleus tractus solitarius
OPCs: oligodendrocyte progenitor cells
pCS: p-Cresol sulfate
PRRs: pattern recognition receptors
PUFAs: polyunsaturated fatty acids
PYY: peptide YY
*RGS4*: regulator of G protein signaling 4 gene
RORγt: receptor-related orphan nuclear receptor gamma t
ROS: reactive oxygen species
RTT: Rett Syndrome
S1DZ: primary somatosensory cortex dysgranular zone
SCFAs: short-chain fatty acids
SNVs: single nucleotide variants
SSRIs: elective serotonin reuptake inhibitors
SV: structural variation
TGFα: transforming growth factor α
Th17 cells: T helper 17 cells
TLR4: toll-like receptor 4
TPH1: tryptophan hydroxylase 1
TRAIL-DR5: tumor necrosis factor-related apoptosis-inducing ligand-decoy receptor 5
VEGF-B: vascular endothelial growth factor B
VNS: vagus nerve stimulation
VTA: ventral tegmental area
